# Generating a robust prediction model for stage I lung adenocarcinoma recurrence after surgical resection

**DOI:** 10.18632/oncotarget.19161

**Published:** 2017-07-11

**Authors:** Yu-Chung Wu, Nien-Chih Wei, Jung-Jyh Hung, Yi-Chen Yeh, Li-Jen Su, Wen-Hu Hsu, Teh-Ying Chou

**Affiliations:** ^1^ Division of Thoracic Surgery, Department of Surgery, Taipei Veterans General Hospital, Taipei, Taiwan; ^2^ Department of Surgery, School of Medicine, National Yang-Ming University, Taipei, Taiwan; ^3^ Auspex Diagnostics, Taipei, Taiwan; ^4^ Division of Molecular Pathology, Department of Pathology and Laboratory Medicine, Taipei Veterans General Hospital, Taipei, Taiwan; ^5^ Institute of Clinical Medicine, School of Medicine, National Yang-Ming University, Taipei, Taiwan; ^6^ Core Facilities for High Throughput Experimental Analysis, Institute of Systems Biology and Bioinformatics, National Central University, Jhong-Li, Taiwan

**Keywords:** lung adenocarcinoma, recurrence, prediction model, data aggregation, adjuvant therapy

## Abstract

Lung cancer mortality remains high even after successful resection. Adjuvant treatment benefits stage II and III patients, but not stage I patients, and most studies fail to predict recurrence in stage I patients. Our study included 211 lung adenocarcinoma patients (stages I–IIIA; 81% stage I) who received curative resections at Taipei Veterans General Hospital between January 2001 and December 2012. We generated a prediction model using 153 samples, with validation using an additional 58 clinical outcome-blinded samples. Gene expression profiles were generated using formalin-fixed, paraffin-embedded tissue samples and microarrays. Data analysis was performed using a supervised clustering method. The prediction model generated from mixed stage samples successfully separated patients at high vs. low risk for recurrence. The validation tests hazard ratio (HR = 4.38) was similar to that of the training tests (HR = 4.53), indicating a robust training process. Our prediction model successfully distinguished high- from low-risk stage IA and IB patients, with a difference in 5-year disease-free survival between high- and low-risk patients of 42% for stage IA and 45% for stage IB (*p* < 0.05). We present a novel and effective model for identifying lung adenocarcinoma patients at high risk for recurrence who may benefit from adjuvant therapy. Our prediction performance of the difference in disease free survival between high risk and low risk groups demonstrates more than two fold improvement over earlier published results.

## INTRODUCTION

Lung cancer patients experience high mortality even after tumor-negative resection, although adjuvant therapy can improve survival. Currently, disease stage is used to guide adjuvant treatment decisions [1]. Adjuvant treatment is recommended for stage II and IIIA patients, and provides measurable survival benefit. Among stage IA and IB patients, only high-risk IB patients are considered for adjuvant treatment, and may receive only marginal benefit [2].

Stage IA and IB non-small-cell lung cancer (NSCLC) patients have 5-year overall survival (OS) rates of only 73% and 54%, respectively [3]. However, studies suggest that adjuvant treatment to ALL stage I patients is detrimental for stage IA and provides no benefit for stage IB [4–6]. Thus, there is a clear need for more accurate recurrence prediction models to identify high-risk stage I patients who could benefit from adjuvant treatment.

### The status of NCSL prognostic publications

Genomic information has been utilized in many studies for recurrence prediction, but is not considered sufficient for clinical consideration [4, 7–9]. In a review of 16 publications [10–25] concerning prognostic clinical factors (e.g. patient selection, tissue handling, etc.), Subramanian, *et al.* noted that most reports did not include patients with stage I disease. In the review’s study design guidelines, the first two study “objectives” should be successful stage IA and IB recurrence predictions. The prediction performance for stage I patients would indicate the quality of the prediction model.

Of these 16 reviewed studies, the Director’s Challenge Consortium (DCC) undertook the largest multicenter study [11]. However, the DCC failed to use genomic information to predict recurrence for stage I patients. Only 2/16 reviewed studies reported stage I results; Potti, *et al.* have since retracted their findings [26], while the study by Lu, *et al.* [23] was a meta-analysis of previously published gene data from different platforms. Subramanian, *et al.* noted that Lu’s prediction model performance was unreliable for stage IA patients; while it demonstrated a survival difference between high- and low-risk patients using training samples, it failed to show a difference in the validation samples. Subramanian, *et al.* attributed these studies’ failures to distinguish between high- and low-risk patients to mathematical errors and clinical design factors. Dupuy, *et al.* found that 50% of published genomic profiling reports had faulty statistical analyses [27]. In summary, all sixteen reviewed studies failed to validate differences in stage IA and IB recurrence predictions. More recently, two new stage I studies with limited performance were published from the University of California, San Francisco (UCSF) and the National Cancer Institute (NCI) [28, 29], against which we will compare our results.

### Issues of meta-analysis studies based on aggregating published gene data

Another issue in interpreting genomic study results stems from the common practice of aggregating published data for re-analysis [12, 14, 18, 22, 23, 30–35]. Most of these studies combine data from different platforms, including different versions of arrays from every major array manufacturer [32], and from PCR analyses, commercial arrays, and custom arrays [12]. Combining gene profiling data is problematic due to the difficulty in reconciling data across different platforms. Such data conversion difficulties were demonstrated in studies comparing several versions of Affymetrix arrays [36–38], which concluded that only genes with similar/identical probes can be compared reliably, in part because arrays from different manufacturers have very different probe designs. This inter-platform issue was studied in a year-long MicroArray Quality Control (MAQC) workshop sponsored by the FDA, which concluded that gene data from different platforms should not be aggregated or compared due to probe sequence and labeling technique differences [39]. A study that used three different platforms to analyze identical RNA samples produced three diverse sets of differentially expressed genes, with only four genes commonly identified across all three platforms [40]. The discovered biomarkers/genes were platform-dependent and not true markers. As a result of these data comparison complications, a data aggregation guideline was proposed [41] and discussed [42]. Not all probes can be converted, and this issue is still under study [43, 44].

Due to the numerous potential issues in an aggregated genomic profiling-based study, external blind validation is essential to confirm a prediction model design. A true validation blinds the clinical outcome of validation samples during model training to avoid possible bias [45]. The validation dataset should be used only once, forcing careful model design and rigorous testing before applying the model to the external, blind validation samples [27]. By this definition, data aggregation studies performed thus far have not undergone true blind validation tests, as all clinical outcomes were already known.

Finally, the data aggregation-based study is an exploratory approach; methods should be repeated in a follow-up study using a chosen platform to validate model performance prior to clinical consideration. So far, even the largest 17-dataset aggregation-based study had only limited performance [34]. We are not aware of any follow-up validation studies based on data aggregation prediction models.

### Considerations of study design

Following the Subramanian, *et al.* guidelines, the present study concentrated on recurrence prediction for stage IA and IB patients for the identification of early stage, high-risk patients to target for adjuvant treatment. Since there are serious potential difficulties in analyzing mixed data from different platforms, we did not use previously published data for validation, but instead generated new data to support both training and validation.

The vast majority of published studies used fresh frozen tissues. If the results were promising, a follow-up formalin-fixed paraffin-embedded (FFPE) based study was carried out for clinical implementation. However, performance and gene selection can vary greatly between fresh frozen and FFPE samples [16, 29]. To prepare for our current study, we ran a pilot study comparing FFPE and fresh-frozen samples. The study included only patients who had paired FFPE and fresh-frozen samples, and the same microarray probes were used for both analyses. The identical patients and probes allowed a detailed comparison between these two results. We found the gene lists chosen for prediction to be vastly different between these two types of tissue preparations. However, prediction performance was similar for fresh-frozen and FFPE samples, and both predicted stage I recurrence successfully. We chose to use FFPE samples for this study.

Many studies use a small number of biomarkers for recurrence prediction, with PCR the most commonly chosen platform. However, due to lung cancer heterogeneity, a large number of genes may be required to provide accurate recurrence predictions. We chose a suitable microarray as the FFPE sample analysis platform, allowing a large number of genes to be screened simultaneously, and providing a stable base to calibrate the values of selected genes for recurrence prediction. By comparison, PCR-based platforms usually only utilize a few genes for calibration.

Many studies chose OS as the primary end point. However, OS was affected by two factors: recurrence and treatment of the recurrence. As the purpose of this study was to reduce recurrence via adjuvant treatment, this study chose disease-free survival (DFS) as the primary endpoint. We also used binary training to force the decision. This encouraged a faster prediction-score transition between high- and low-risk predictions. A prediction-score curve typically transitions smoothly between high- and low-risk ranges, but an “intermediate risk” transitional group is less useful in clinical decision making. Finally, to ensure the accuracy of our prediction model, external blind validation was implemented. This forced a careful training process to ensure that no bias was introduced in the model design.

## RESULTS

**Figure 1:**
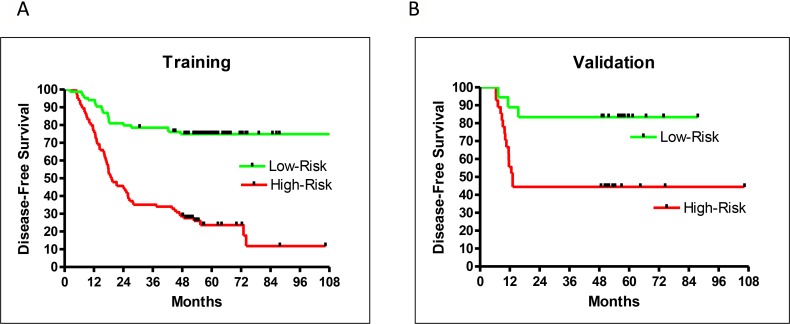
Prediction performance by DFS for training (**A**), and validation (**B**) samples.

Of the 211 patient samples included in this study, 153 were used for training, and the remaining 58 for blind testing. During training, a leave-5-out method was used to randomly assign five samples to be self-testing with the remaining 148 used for training. This training process was repeated 500 times to form an averaged prediction performance with 102 selected genes (Supplementary File: Gene List). The hazard ratio (HR) of recurrence for high- vs low-risk groups from the training set was very good at 4.53 (95% confidence interval (CI): 2.77–6.35, *P* < 0.0001). DFS rates were well separated between high- and low-risk patients five years after surgery (51% difference) (Figure 1A).

An additional 58 clinical outcome-blinded samples were used for external validation. A small *p*-value (< 0.05) for separation of the predicted high- and low-risk validation samples confirmed the performance of the prediction model. However, this did not necessarily indicate a similar performance between training and validation. Only a careful and unbiased training procedure allowed validation samples to achieve a performance similar to that of the training samples. This study achieved similar training and validation performances. The HR of recurrence for high- vs low-risk groups from the validation samples was 4.38 (95% CI: 1.34–8.63, *P* = 0.0101; Figure 1B), which was very close to the HR of recurrence (4.53) from the training set. Both training and validation had excellent 5-year DFS separation between high- and low-risk patients (Figure 1A–1B).

**Figure 2:**
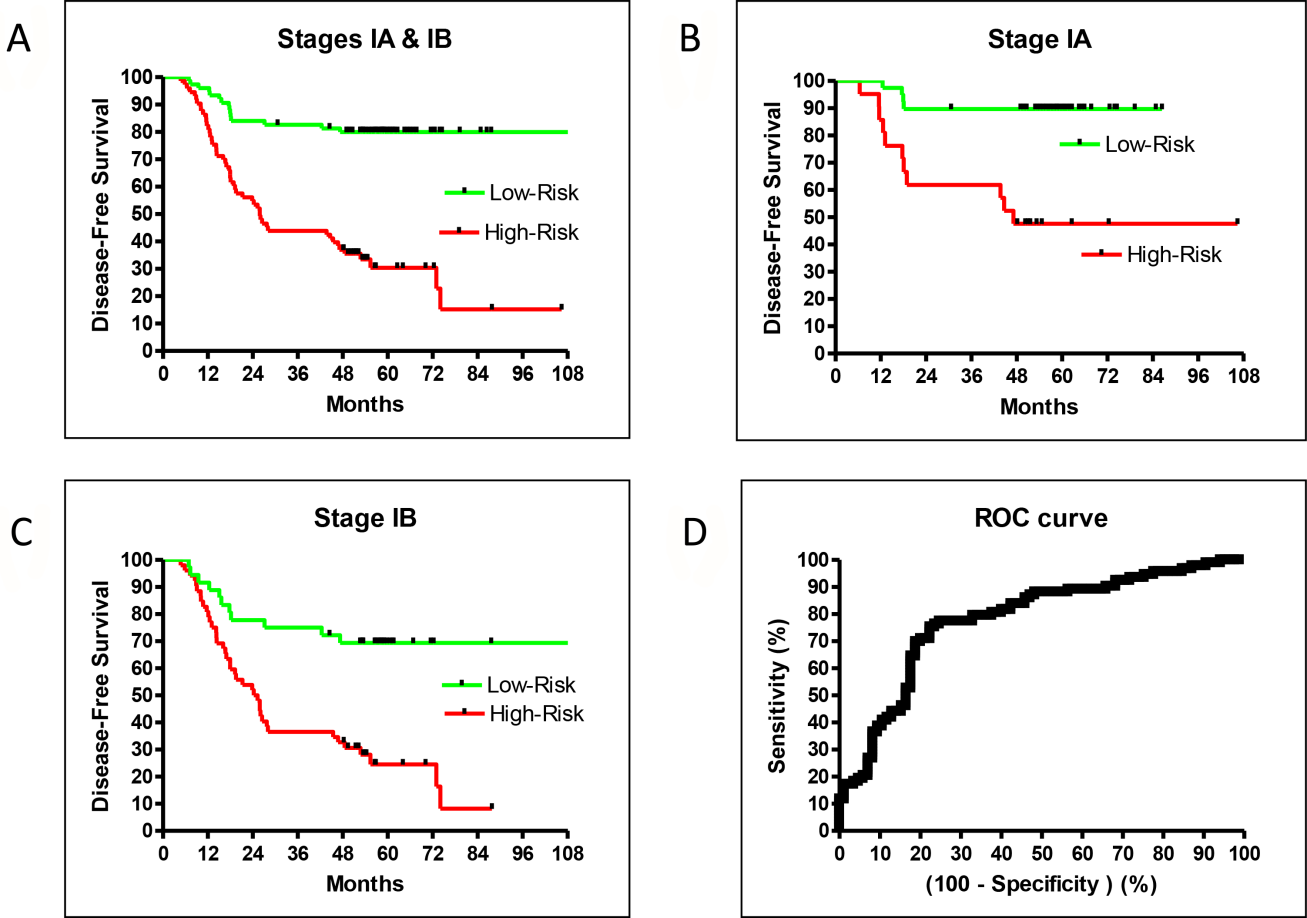
DFS in the training set for all stage IA + IB (**A**), stage IA alone (**B**), stage IB alone (**C**), and ROC (**D**).

The validation sample set confirmed the recurrence prediction model’s excellent overall performance. While there were not enough validation samples to test stage IA and IB patients separately, the validation and training performances were very similar and therefore a close indication for the separate performances for stage IA and IB patients. The HR of high- vs low-risk recurrence of stage I-only training samples was 4.78 (95% CI: 2.78–7.48, *P* < 0.0001), similar to the training result for patients of all stages (HR = 4.53; Figures 1A and 2B). Additionally, the prediction model separated high- and low-risk patients for stage IA or IB cases. The HR of recurrence for high- vs low-risk stage IA patients was 6.39 (95% CI: 2.61–23.8, *P* = 0.0003), and the 5-year DFS difference between high- and low-risk patients was 42% (Figure 2B). The HR of recurrence for high- vs low-risk stage IB patients was 3.46 (95% CI: 1.74–5.28, *P* < 0.0001), while the 5-year DFS difference between high- and low-risk patients was 45% (Figure 2C). While both groups were difficult to predict, the prediction model performed well for both sets of patients.

Our model was trained by weighing recurrence and non-recurrence errors equally. This led to a balanced performance between sensitivity and specificity. Using a default cutoff value of zero, the prediction model had a sensitivity = 0.77 and a specificity = 0.74. If higher sensitivity was preferred, one could retrain using larger error weights for recurrent samples. As the area under the curve (AUC) of the receiver operator characteristic (ROC) of this prediction model was good at 0.78 (95% CI: 0.71–0.85, *P* < 0.0001; Figure 2D), one could trade an excellent sensitivity and still have a reasonable specificity using different cutoffs without retraining (e.g. sensitivity at 0.90 and specificity at 0.51).

### Identification of high-risk stage I patients

The sensitivity and specificity of our predictive model allowed for the clear separation of high- and low-risk patients from the average recurrence rate. For example, low-risk IA patients had a 5-year DFS rate of about 90%, while high-risk IA patients had a rate of about 50% (Figure 2B). A large survey reported a 73% 5-year OS for stage IA patients [3], which was about the middle of the high- and low-risk values. Similarly, the low-risk IB patients had a DFS rate of about 70% while high-risk IB patients had a rate of about 30% (Figure 2C). The reported 5-year OS for stage 1B patients was 58% [3], which was also in the middle of our high- and low-risk rates. Therefore, our prediction model successfully identified high-risk stage 1A and 1B patients for adjuvant treatment.

## DISCUSSION

### Marker gene discovery vs. phenotypic classification

Many recent studies have aggregated published genomic data from different platforms to train or validate their prediction models. Since the data were not fully compatible among different platforms, the act of data aggregation implied an assumption that the identified biomarkers were true marker genes that could overcome platform differences. It thus became common practice to validate discovered gene lists via several published datasets. In short, the marker gene concept was implicitly included in data aggregation studies even when the concept was not stated. Some data aggregation studies explicitly included the concept of marker genes, as pathway information was used to select potential genes [22]. One study used prostate cancer patients to identify recurrence-related genes, then extrapolated to predict recurrence in lung cancer patients [46]. Thus, marker gene concepts were implicitly or explicitly included for all data aggregation studies.

The marker gene concept has also lead to many studies using only one gene to predict recurrence [8]. As NSCLC is known for its heterogeneity, with different survival rates associated with different subtypes [10, 47], it is not surprising that a large number of single gene studies have failed to predict recurrence [8].

Marker gene discovery is often a search for one or two important genes. This requires accurate expression values for all genes to avoid missing the target gene(s), thus necessitating the use of fresh-frozen tissues for analysis. As fresh frozen tissue collection is not a standardized procedure, sample quality can vary greatly among different clinics. Conversely, FFPE tissue collection is standardized, with similar quality across different institutions, and therefore excellent translational potential.

Marker gene discovery and phenotypic classification are two different tasks with different requirements: gene expression accuracy vs. consistency. For gene discovery, data accuracy from every gene is key. For recurrence prediction, gene data consistency is integral for prediction accuracy from multiple genes. For this reason, FFPE tissue could be a better choice for the current task. The present study demonstrates the potential for using FFPE tissue in clinical practice.

There is inherent risk in using a large number of genes for recurrence prediction without careful design. Bias could easily be introduced due to the large number of screened candidate genes compared to the much smaller number of patient samples. A true validation set forces a careful training system design to ensure no bias is introduced from using a larger number of genes. Our careful training procedure allowed the validation set to achieve a similar performance as the training set.

### Comparison to other stage I studies

Most recent studies have re-analyzed already-published data sets. Only two groups, the NCI [28, 33] and UCSF [16, 29], that predicted stage I recurrence used newly processed samples. Both studies used fresh-frozen tissues and analyzed gene expression using PCR. In the UCSF study, FFPE samples were used in a follow-up study. In fresh-frozen vs. FFPE samples, the genes selected for prediction increased from 4 to 11; however, model performance was negatively affected. The HR for the training sets was reduced from 6.72 for stage I–III patients [16] to 2.43 (stage I), 2.68 (stage II), and 1.93 (stage III) [29].

A direct comparison of our study with that of the UCSF group was difficult due to OS (UCSF) vs DFS (this study) endpoint differences. Additionally, UCSF had three prediction classifications (high-, intermediate-, and low-risk) with equal population distribution. The removal of intermediate-risk patients improves the HR of distinguishing high- vs low-risk. As an intermediate-risk classification limits its use in clinical decision-making, we used binary prediction classifications (high- vs low-risk). Still, a qualitative comparison between the UCSF model and this study was possible; the Kaiser testing set from UCSF had a high- vs low-risk OS HR of 2.16. The validation result was close to training result, with HR=2.43. The 5-year OS difference between high- vs. low-risk patients was 18.4%.

The NCI study also differed from our study, using PCR to analyze gene expression in fresh frozen tissue. NCI did not follow up with an FFPE study, thus preventing a direct comparison with our FFPE-based results. The NCI trained their model using DFS, but the validation was done using OS from nine published datasets generated from different platforms [33]. NCI also used a 3-class prediction vs. our 2-class prediction. However, we were still able to qualitatively compare our study with theirs. The HR for high- vs low-risk DFS from the stage I NCI training set was 2.19. The HR for high- vs low-risk OS from nine different validation datasets was 1.73. The 5-year OS difference between high- and low-risk patients was estimated at 25% [33]. A follow-up FFPE study is necessary for translation to clinical implementation; tissue preparation type changes can greatly affect performance as shown by the UCSF study results.

Instead of three class predictions, our study used binary classifications, i.e. all patients were included in the HR calculation. The 5-year DFS difference between low-risk and high- stage I patients was 49.5% (79.9% vs. 30.4%) with HR = 4.87. Within the recognized limitations of study design differences, comparing these values to the UCSF and NCI results, our 5-year DFS difference between high- and low-risk patients is estimated to be about twice as large as previously reported.

In addition, we observed excellent separation and HR between high- and low-risk stage IA and IB patients. The difference was 42.1% (89.7% vs. 47.6%) and HR was 6.40 for stage IA patients, while the difference was 44.7% (69.3% vs. 24.6 %) and HR was 3.46 for stage IB patients. As it is the most difficult to predict recurrence for stage IA patients, the excellent HR for stage IA is a good indicator of the robustness of this prediction model.

We compared the gene list identified by the NCI (4 genes) and UCSF (11 genes) to our list (102 genes). None of the NCI and UCSF target genes were on our list. If the small number of genes discovered by the NCI and UCSF are true marker genes, our study shows that one does not need marker genes for good recurrence prediction. This further supports the observation that gene lists are platform dependent.

## MATERIALS AND METHODS

### Patients

Medical charts of patients who had undergone lung adenocarcinoma curative resections between January 2001 and December 2012 were reviewed and selected by a 1:1 ratio based on recurrence or non-recurrence status. This ratio allowed the prediction model to have a balanced performance for recurrence and non-recurrence patients. A total of 101 non-recurrent and 110 recurrent patients with sufficient FFPE samples from the Taipei Veterans General Hospital tissue bank were enrolled in this study. During the last five decades the hospital has evolved into a medical center that caters to the general public as well as to veterans. This patient population provided a roughly balanced male to female ratio for this study. The study was approved by the Institutional Review Board of Taipei Veterans General Hospital (IRB Number: 2013-06-005AC). Written informed consent was obtained from all patients.

Median follow-up time was 53.2 months. Non-recurrent patients had a minimum follow-up of 48 months. A majority of these (81%, 171/211) were stage I patients (Table 1, Table 2A, Table 2B). By including a smaller number of stage II and III samples, the training procedure selected more robust genes across different stages to form the prediction model. None of the 101 non-recurrent patients received adjuvant treatment, while 29/110 recurrent patients did.

**Table 1:** Characteristics of all patients with or without recurrence

Variables	Non-Recurrence (*N* = 101)	Recurrence (*N* = 110)
Age, years (median ± SD)	63.4 ± 11.8	68.0 ± 11.7
Follow-up, months (median ± SD)	56.8 ± 12.7	41.7 ± 19.0
Sex, number (%)		
Male	51 (50.5)	60 (54.5)
Female	50 (49.5)	50 (45.5)
Stage, number (%)		
IA	50 (49.5)	18 (16.4)
IB	48 (47.5)	55 (50.0)
IIA	1 (1.0)	10 (9.1)
IIB	1 (1.0)	4 (3.6)
IIIA	1 (1.0)	23 (20.9)

SD, standard deviation.

**Table 2A:** Comparison of training and validation patients without recurrence

Non-Recurrence (*N* = 101)	Training (*N* = 73)	Validation (*N* = 28)
Age, years (median ± SD)	62.0 ± 10.6	68.5 ± 15.5
Follow-up, months (median ± SD)	56.8 ± 12.1	55.9 ± 12.2
Sex, number (%)		
Male	36 (49.3)	15 (53.6)
Female	37 (50.7)	13 (46.4)
Stage, number (%)		
IA	41 (56.2)	9 (32.1)
IB	30 (41.1)	18 (64.3)
IIA	1 (1.4)	0 (0.0)
IIB	1 (1.4)	0 (0.0)
IIIA	0 (0.0)	1 (3.6)

SD, standard deviation.

**Table 2B:** Comparison of training and validation patients with recurrence

Recurrence (*N* = 110)	Training (*N* = 80)	Validation (*N* = 30)
Age, years (median ± SD)	68.5 ± 11.9	66.0 ± 11.4
Follow-up, months (median ± SD)	47.2 ± 20.0	34.1 ± 13.9
Sex, number (%)		
Male	44 (55.0)	16 (53.3)
Female	36 (45.0)	14 (46.7)
Stage, number (%)		
IA	10 (12.5)	8 (26.7)
IB	44 (55.0)	11 (36.7)
IIA	8 (10.0)	2 (6.7)
IIB	4 (5.0)	0 (0.0)
IIIA	14 (17.5)	9 (30.0)

SD, standard deviation.

Complete tumor resection combined with mediastinal lymph node dissection or sampling was performed in all patients as previously described [48, 49]. Disease stages were determined based on TNM classification (7th ed.) of the American Joint Committee on Cancer and the International Union Against Cancer. All patients were followed up at the outpatient department in 3-month intervals for the first two years after resection and in 6-month intervals thereafter. Patient OS rate was calculated from the date of operation to the date of event (death). DFS was defined as the time between surgery and the occurrence of an event (death or recurrence). Censored data are that when an event did not occur, and survival time was calculated from surgery to the date of last follow up.

### Platform

Affymetrix GeneChip^®^ Human ST 2.0 microarray was used for this study. ST 2.0 is a whole-transcript array that includes probes to measure 40,716 RefSeq transcripts and 11,086 long intergenic non-coding RNA transcripts (lincRNA). The array contained more than 1.35 million probes distributed across the full length of genes, providing an excellent measurement of overall gene expression for FFPE samples.

### Data generation

For each sample, a 10-μm FFPE section was used to extract total RNA using Qiasymphony automation with the Qiasymphony RNA Kit from Qiagen. Samples were fragmented and labeled using the NuGEN Encore Biotin kit according to the manufacturer’s specifications. Hybridization cocktails containing 3.75 ug of the fragmented, end-labeled cDNA were applied to GeneChip^®^ Human Gene 2.0 ST arrays. Hybridization was performed for 17 h, and arrays were washed and stained with the GeneChip Fluidics Station 450 using FS450_0007 script. Arrays were scanned using the Affymetrix GCS 3000 7G and GeneChip Operating Software v. 1.4 to produce CEL intensity files. The complete dataset GSE90623 can be accessed at NCBI’s Gene Expression Omnibus (GEO) .

### Data analysis

Gene data and quality control metrics were extracted from Cell Intensity File (CEL) using Affymetrix software Affymetrix Power Tools (APT). Robust Multi-array Average method was used for normalization. Hybridization process quality was monitored using Affymetrix bacterial spikes, and labeling process quality was monitored with poly-A-control RNAs. The metrics of each sample had to be within the vender’s quality specifications; otherwise the entire process was repeated.

To ensure the stability of our chosen platform, samples were extracted over 20 batches to test gene expression variation between batches; a reference sample was added to each sample-processing batch. Reference samples from different batches were compared to ensure data consistency across different batches.

A k-Nearest Neighbors (KNN) algorithm was used to build a prediction model to differentiate recurrence from non-recurrence samples. An unknown sample was classified as recurrent or non-recurrent, based on the classification of its nearest neighbor. The distance metric was the correlation of gene expression between samples. A *t*-test was used to select the best genes to calculate correlation.

The prediction model was trained to have a maximum distinction between two classifications: recurrent/high-risk or non-recurrent/low-risk samples. After training, the prediction model output a score for each test sample. The model had a default cutoff value of zero to separate recurrent (> 0) and non-recurrent (< 0) patient predictions. A negative score indicated a low-risk prediction; a positive score indicated a high-risk prediction. A larger absolute score signified a prediction with high confidence; low confidence prediction scores (–0.5 < score < 0.5) were considered non-decision/medium-risk cases. The score allowed a tradeoff between sensitivity and selectivity using different cutoffs.

### Training and external validation

During training, 153 training samples were randomly separated into two groups; 148 samples were used to generate the prediction model, while five were reserved to test model performance. These two sample groups were well separated in the computer programming to simulate the final external validation test with an additional 58 samples. Careful repetitions of this simulated test ensured the subsequent success of the validation test.

To implement a blind validation process, the clinical outcomes of the 58 test samples were unknown during the training procedure to avoid bias. Only after the model had generated the 58 test sample predictions were clinical outcomes compared to predictions. This procedure ensured that the 58 samples provided true external validation.

### Performance indicators

The recurrence prediction model performance was indicated by the HR of recurrence between predicted high- vs. low-risk patients. AUC under the ROC curve, sensitivity, and specificity were also reported. GraphPad PRISM was used to generate the results.

## CONCLUSIONS

As the prediction results of most published studies using marker genes have been limited, we discarded the idea of identifying marker gene lists and used phenotypic classification instead. To build a successful classification-based predictive model, we generated a new high quality gene expression dataset from FFPE samples using an automated process, thus avoiding the hazards of utilizing published data from different platforms for validation purposes. During analysis, the integrity of the prediction model was rigorously tested and the performance validated using a blind data set. The use of consistent and high quality data combined with rigorous iterative training resulted in successful recurrence predictions for both stage IA and IB patients, and suggested the possibility of excellent clinical performance using this new approach. The prediction performance of our model was improved more than two-fold compared to previously published results.

Stage I patients are currently a small percentage of all lung cancer patients. With the recent recommendation of using low dose CT for lung cancer screening, stage I patient detection will increase. We present a novel and efficacious model that identifies high-risk stage I lung cancer patients who may benefit from adjuvant treatment, and therefore may improve patient survival.

## SUPPLEMENTARY MATERIALS




