# Supplementary material for: Generating a robust prediction model for stage I lung adenocarcinoma recurrence after surgical resection
Source: Oncotarget. 2017 Jul 11;8(45):79712–21. doi: 10.18632/oncotarget.19161 (PMC5668084; doi:10.18632/oncotarget.19161)
Supplement: Supplementary file 1 [file oncotarget-08-79712-s001.pdf]

Generating a robust prediction model for stage I lung adenocarcinoma recurrence after surgical resection

SUPPLEMENTARY MATERIALS

Supplementary-GeneList: See_Supplementary-GeneList.
